# Harnessing Advanced Machine Learning Techniques for Microscopic Vessel Segmentation in Pulmonary Fibrosis Using Novel Hierarchical Phase-Contrast Tomography Images

**DOI:** 10.1055/a-2540-8166

**Published:** 2025-05-09

**Authors:** Pardeep Vasudev, Mehran Azimbagirad, Shahab Aslani, Moucheng Xu, Yufei Wang, Robert Chapman, Hannah Coleman, Christopher Werlein, Claire Walsh, Peter Lee, Paul Tafforeau, Joseph Jacob

**Affiliations:** 1 Institute of Health Informatics, Faculty of Population Sciences, University College London, London, United Kingdom; 2 Centre of Medical Image Computing, University College London, London, United Kingdom; 3 Department of Mechanical Engineering, University College London, London, United Kingdom; 4 Division of Medicine, University College London, London, United Kingdom; 5 Centre for Advanced Biomedical Imaging, University College London, London, United Kingdom; 6 Institute of Pathology, Hannover Medical School, Hannover, Germany; 7 European Synchrotron Radiation Facility, Grenoble, France; 8 UCL Respiratory, University College London, London, United Kingdom

**Keywords:** vessel segmentation, hierarchical phase-contrast tomography, semi-supervised learning, pulmonary fibrosis, interstitial lung disease

## Abstract

**Background** Fibrotic lung disease is a progressive illness that causes scarring and ultimately respiratory failure, with irreversible damage by the time it is diagnosed on computed tomography imaging. Recent research postulates the role of the lung vasculature on the pathogenesis of the disease. With the recent development of high-resolution hierarchical phase-contrast tomography (HiP-CT), we have the potential to understand and detect changes in the lungs long before conventional imaging. However, to gain quantitative insight into vascular changes you first need to be able to segment the vessels before further downstream analysis can be conducted. Aside from this, HiP-CT generates large-volume, high-resolution data which is time-consuming and expensive to label.

**Objectives** This project aims to qualitatively assess the latest machine learning methods for vessel segmentation in HiP-CT data to enable label propagation as the first step for imaging biomarker discovery, with the goal to identify early-stage interstitial lung disease amenable to treatment, before fibrosis begins.

**Methods** Semisupervised learning (SSL) has become a growing method to tackle sparsely labeled datasets due to its leveraging of unlabeled data. In this study, we will compare two SSL methods; Seg PL, based on pseudo-labeling, and MisMatch, using consistency regularization against state-of-the-art supervised learning method, nnU-Net, on vessel segmentation in sparsely labeled lung HiP-CT data.

**Results** On initial experimentation, both MisMatch and SegPL showed promising performance on qualitative review. In comparison with supervised learning, both MisMatch and SegPL showed better out-of-distribution performance within the same sample (different vessel morphology and texture vessels), though supervised learning provided more consistent segmentations for well-represented labels in the limited annotations.

**Conclusion** Further quantitative research is required to better assess the generalizability of these findings, though they show promising first steps toward leveraging this novel data to tackle fibrotic lung disease.

## Introduction

The National Health Service (NHS) aims to improve lung cancer detection, by expanding the Lung Health Check program for individuals aged 55 to 74 with a General Practice (GP) record and a history of smoking. 1 High-risk individuals will receive a CT scan every 2 years, the standard method for lung cancer detection in the United Kingdom. 2 Currently available in limited United Kingdom centers, the NHS aims for full coverage by 2029, increasing annual CT scans from 200,000 to 1 million. 3 Approximately 2% of cases will have lung cancer, and approximately 1.5% will have pulmonary fibrosis. 4

Pulmonary fibrosis describes the presence of lung thickening and scarring, resulting in symptoms such as shortness of breath and a cough. 5 It can be the result of a variety of heterogeneous conditions that can have varying prognosis, including interstitial lung diseases (ILDs) such as idiopathic pulmonary fibrosis (IPF) and pleuroparenchymal fibroelastosis (PPFE), which have median survival times of 2.5 to 5 years. 6 7 IPF is characterized by progressive scarring of the lungs with alveolar destruction, leaving the lungs stiff with decreasing ability for gaseous exchange, causing significant morbidity and eventually respiratory failure, with a median survival of 3 to 4 years without treatment. 8 By contrast, PPFE is a relatively rare ILD that involves scarring of the upper lobes involving the pleura and subpleural lung. 9 The prognosis varies depending on the phenotype, though is worse for those with preexisting IPF and may be worse in the late stages of idiopathic PPFE than IPF. 6 10 11 Early-stage disease is often asymptomatic or presents with non-specific symptoms, such as progressive shortness of breath, cough, and lethargy. 12 Currently, pulmonary fibrosis is best assessed on CT, though visual analysis methods were designed for the description of imaging patterns constituting established and irreversible disease. Hence, sensitive and specific descriptors of early-stage are not well-known due to a lack of corresponding histopathological scale ground truth with which to base clinical CT descriptors, limiting treatment options to supportive care or, more recently, antifibrotic agents that target patients in the late stages of the disease. 13

One of the main challenges in identifying early-stage imaging biomarkers is the lack of understanding of the exact pathophysiological mechanisms of both diseases. Some studies suggest that microvascular changes in fibrotic lung tissues may not only be a result but also a cause of these lung conditions with abnormal anastomoses (connections between the pleural and parenchymal blood circulation), vascular remodelling, and capillary dilatation all seen. 8 14 15 Hence, understanding the nature of the vasculature in both healthy individuals and those with established PPFE or IPF may enable the discovery of specific imaging biomarkers to identify those at risk of progressive disease. This information could also then be used not only to identify patients for possible drug trials and allow for a method of monitoring their progress but also as targets for therapies aimed at vascular remodelling and angiogenesis.

### Hierarchical Phase-Contrast Tomography

Hierarchical phase-contrast tomography (HiP-CT), a novel three-dimensional imaging method using an X-ray propagation technique, offers greater resolution and precision in ex vivo imaging. 16 HiP-CT advances tissue differentiation by utilizing phase contrast imaging, relying on the phase shift of X-rays passing through different tissues, achieving greater resolution at the microscopic scale compared with attenuation-based X-ray imaging. 17 Its hierarchical approach uses phase-contrast imaging scans at varying scales, providing anatomical structure visualization from organ-level overviews to microscopic details, achieving down to 2.5-μm resolution. 16 Despite its promise, HiP-CT faces challenges, primarily the vast data volumes it generates with a single volume of interest through lung depth captured at 6 μm per voxel amassing approximately 600 GB of data. 16

### The Challenge of Sparsely Labeled Data

Given the potential ability to assess microvascular changes on HiP-CT, an initial step would be to identify a method to segment the vasculature on HiP-CT for further quantitative insight. A recent review on blood vessel segmentation concluded that factors beyond model choice, such as varied metric choices, lack of definite ground truth, and insufficient study of pathological vessels, make it difficult to define a “gold standard algorithm.” 18 Supervised learning techniques require large volumes of high-quality labeled data to accurately represent the data distribution for optimal model performance. 19 Without this, issues such as overfitting and lack of generalizability can arise. In this study, and generally in medical imaging, obtaining sufficient high-quality labeled data is challenging due to several factors, primarily the high time and monetary costs of expert labeling, resulting in sparsely labeled or small datasets. These datasets present several challenges, including bias due to data imbalance, as sparse labels may lead to certain features being labeled more frequently. 20 For example, in vessel segmentation, larger and well-defined vessels may be annotated more often than smaller ones, leading to better quality segmentation for larger vessels. Additionally, less skilled or experienced labelers may be used, resulting in decreased annotation quality and less reliable data. 21 Finally, the same feature might be labeled inconsistently by the same labeler (intraobserver variability) or different labelers (interobserver variability), hindering the consistency of labels. 19 While supervised learning with labeled data remains the “gold standard,” semisupervised learning (SSL) aims to tackle label scarcity by leveraging unlabeled data.

### Semisupervised Learning

SSL harnesses both supervised and unsupervised learning by using labeled and unlabeled data to make predictions. 22 It is particularly useful for small datasets or sparse labels as it leverages large amounts of unlabeled data, reducing the need for extensive labeled data. 23 In this study, we use semisupervised methods based on consistency regularization, derived from entropy minimization to reduce prediction uncertainty as a strong regularization technique on unlabeled data to find a decision boundary. 24 25 Consistency regularization can be thought of as having two different types, soft and hard. 25 Soft regularization applies a distance-based loss function to predicted probabilities, allowing for similar but not identical predictions. Hard regularization uses pseudo-labels with strict boundaries to train model predictions. Of note, both methods can be used at the input level and feature level.

The soft concept of consistency regularization was first proposed by Bachman et al, and subsequently popularized through the introduction of the Pseudo-Ensemble Agreement regularization, a term that is used to minimize the difference between the output of an original data point (so-called parent) and its perturbed versions (so-called children). 26 27 28 In essence, when a parent data point is perturbed, it creates several children's data points. Applying the regularization term to these children's data points ensures they align on the same lower-dimensional manifold or “surface” within a higher-dimensional space, thus producing similar outputs. This approach effectively utilizes unlabeled data, which, although not explicitly labeled, now carries useful information that can be leveraged. Consequently, it helps a model produce consistent outputs when given the same input subjected to different semantic-preserving perturbations. The sensitivity to perturbations causing differences in predictions on the same input is penalized by a regularization term, typically based on mean square error or K-L divergence.

Another methodology to leverage unlabeled data in the chosen models is the use of pseudo-labels, a long-standing concept popularized by Lee. 29 The idea is for the network to initially train on the available labeled data, which is then used to make predictions on the unlabeled data. If a certain confidence threshold is met, the predicted label is treated as a “‘pseudo-label” for the unlabeled data, which is then incorporated into the labeled dataset for subsequent training iterations. The threshold is crucial to ensure that the predicted pseudo-labels are accurate, as one significant limitation of this technique is the potential propagation of errors if the predicted labels are incorrect. 30 A subsequent state-of-the-art technique, FixMatch, proposed a streamlined approach that combines both consistency regularization at the input level and pseudo-labeling. 31 In this method, a weakly augmented image is first fed into the model, and pseudo-labels are generated based on the model's predictions. If the model produces a high-confidence prediction above a certain threshold for a given image, that pseudo-label is retained. Then, when the model is presented with a strongly augmented version of the same image, it is trained to predict the pseudo-label using cross-entropy loss. These models form the foundation of the current models being used in the project.

This study employs two state-of-the-art SSL algorithms. The first, MisMatch uses morphological perturbations at the feature level with consistency regularization, learning optimal perturbations from data via attention mechanisms. 32 The second, SegPL, is a purely pseudo-label-based model set as an expectation maximization problem, offering robust performance against noise and adversarial attacks with less computational cost. 25 33

Both models outperform existing state-of-the-art models, including FixMatch, when applied to pulmonary vessel segmentation, although this is on traditional CT imaging.

### Objectives

To achieve this, the project aims to evaluate the chosen SSL methods for segmenting vasculature in HiP-CT datasets. The goal is to identify histopathology correlating imaging biomarkers of early fibrosis that could be amenable to potential pharmaceutical intervention. Additionally, the performance of these semisupervised models will be compared against the state-of-the-art supervised learning technique nnU-Net to assess improvements in handling label-scarce environments. This project is the initial step into understanding challenges in producing optimal segmentations of the vasculature in novel HiP-CT data to ultimately discover new biomarkers in early fibrosis.

#### Research Question

How effective are the latest machine learning techniques (e.g., SSL and nnU-Net) in performing vessel segmentation on novel HiP-CT images with sparse annotations to delineate vascular anatomy in cases of early pulmonary fibrosis?

## Methodology

### Dataset Selection and Curation

The data for this study comprised HiP-CT images of the lungs from the European Synchrotron Radiation Facility Extremely Brilliant Source (ESRF-EBS), with lung tissues scanned at 25-μm voxel resolution, with regions of interest further zoomed to 6- and 1–2.5-μm resolution—over 100 times the resolution of current clinical CT. The datasets used in this study comprised “substacks” of 2.5-μm resolution from lung biopsy samples, representing a small portion of the total imaged volume.

**Fig. 1:**
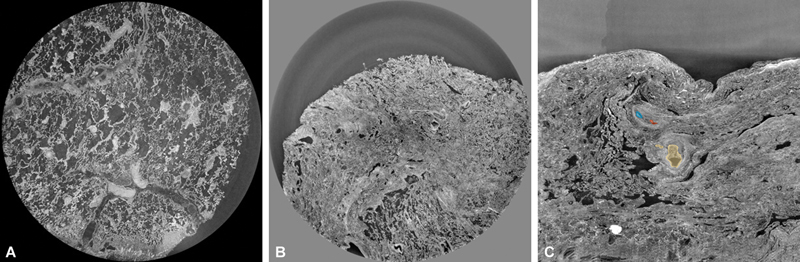
Example of 2.5-μm resolution Synchrotron data of the lung. **(A)** Biopsy sample with PPFE. **(B)** Biopsy sample with PPFE and IPF. **(C)** Sparsely annotated sample with PPFE and IPF. IPF, idiopathic pulmonary fibrosis; PPFE, pleuroparenchymal fibroelastosis.

The original substacks came from two different sources: an area with PPFE ( Fig. 1A ), and an area with PPFE in a patient with IPF ( Fig. 1B ). The PPFE/IPF sample consisted of 960 slices with dimensions of 1823 × 1823 pixels and 1 mm spacing between slices (dataset C). The PPFE sample delivered two substacks: one with 1838 × 1838 pixels, 1,863 slices, and 1 mm spacing (Dataset A), and another with 1838 × 1838 pixels, 220 slices, and 1 mm spacing (Dataset B), which only became available later in the study. The largest substack was over 6 GB when stored in Neuroimaging Informatics Technology Initiative (NIfTI) format and zipped.

Annotations were manually performed to label the vessels, including both the vessel wall and the lumen. The labeling process was a group consensus effort involving consultation with a consultant thoracic radiologist and arbitration by a pathologist for uncertain cases. Non-experts performed the actual labeling individually for each of the three available datasets. Given the large volume of data, a recurrent cadence was used, with a growing algorithm in 3D Slicer to interpolate the vessel between slices. Vessels were identified by drawing around the vessel and filling the outlined region ( Fig. 1C ).

An additional unlabeled dataset from the PPFE-only source was used for training for the semisupervised models, consisting of 1,300 slices of 1823 × 1823 pixels (Dataset D).

### Models

#### MisMatch

MisMatch 32 is a method that improves semisupervised segmentation by perturbing morphological features of unlabeled images with consistency regularization. The model leverages different attention mechanisms to, respectively, dilate and erode foreground features that are combined in a consistency-driven framework. Any encoder–decoder architecture can accommodate the MisMatch framework.

**Fig. 2:**
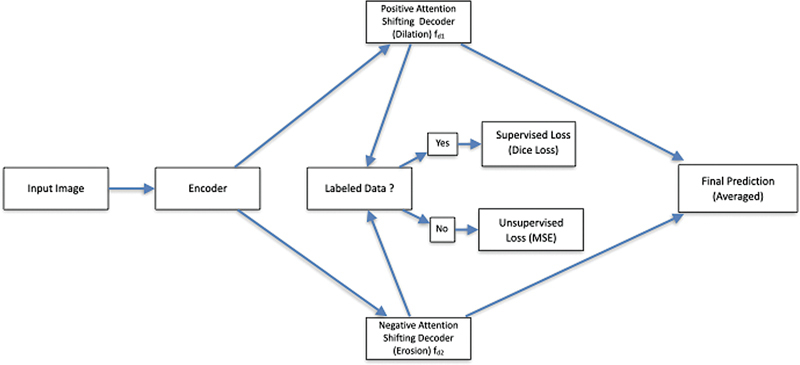
Workflow of the MisMatch framework for semisupervised segmentation. The encoder processes the input image, generating features that are passed to two parallel decoders: the positive attention shifting decoder (  ) for dilated feature prediction and the negative attention shifting decoder (  ) for eroded feature prediction. For labeled data, supervised loss (Dice Loss) is calculated, while for unlabeled data, consistency regularization (MSE Loss) is applied between the outputs of  and  . The final segmentation prediction is obtained by averaging the outputs of the two decoders.

MisMatch is a semisupervised segmentation method designed to leverage unlabeled data through morphological perturbations of feature maps. The approach combines dilation and erosion operations with consistency regularization, effectively manipulating the effective receptive field (ERF) of the network's predictions to enhance segmentation performance. The MisMatch architecture consists of an encoder–decoder framework with two parallel decoder branches that apply distinct attention-shifting mechanisms ( Fig. 2 ).

At the heart of MisMatch is the concept of the ERF, which measures the region of an image contributing most significantly to the prediction of a central pixel. By controlling the ERF, the framework enhances the model's ability to differentiate between foreground and background features. Larger ERFs, achieved through dilated convolutions, allow for high-confidence predictions over broader regions, simulating dilation. Conversely, smaller ERFs, facilitated by skip connections, restrict the model's focus to a narrower context, mimicking erosion.

The architecture comprises a single encoder, *f*_e_ , which extracts high-dimensional feature maps from the input image. These feature maps are then fed into two parallel decoders:

##### Positive Attention Shifting Block

This decoder focuses on expanding the ERF using dilated convolutions with a dilation rate of 5. The outputs from the main branch and the dilated side branch are combined using element-wise multiplication to enhance foreground predictions.

##### Negative Attention Shifting Block

This decoder reduces the ERF through skip connections, which ensemble shorter effective paths. The outputs from its main and side branches are also combined element-wise to simulate the erosion of the feature map.

The outputs of the positive attention shifting block (PASB) and negative attention shifting block (NASB) are subsequently averaged to produce the final segmentation prediction. This design ensures a balanced perspective, capturing both dilated and eroded features.

The training process for MisMatch employs distinct loss functions depending on the data type:

For labeled data, the supervised loss is computed using the Dice coefficient:



For unlabelled data, consistency regularization loss is applied between the outputs of the two decoders:



The total loss function combines these two components, weighted by a hyperparameter α\αα to balance supervised and unsupervised learning:



Fig. 2 provides a visual representation of this workflow, showing the parallel paths from the encoder to the PASB and NASB, the merging of their outputs, and the delineation of supervised and unsupervised loss calculations. As depicted, labeled data follow a path to supervised Dice loss computation, while unlabeled data proceed to the consistency regularization stage, reflecting the dual objectives of the framework.

By integrating these components, MisMatch effectively captures the complementary benefits of dilation and erosion, ensuring robust segmentation even in scenarios with limited labeled data. The framework's flexibility allows it to be applied to various encoder–decoder architectures, making it a versatile choice for semisupervised segmentation tasks.

#### SegPL (Bayesian Pseudo-Labels)

**Fig. 3:**
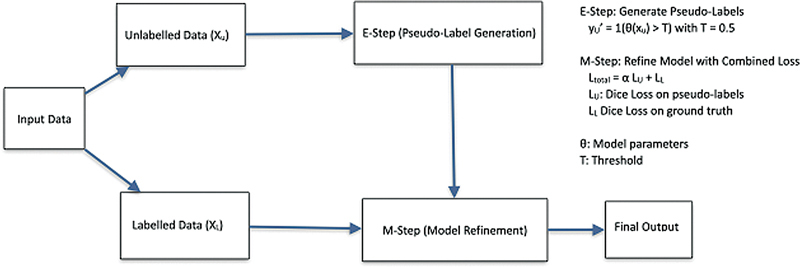
Workflow of the SegPL algorithm with a fixed threshold for pseudo-label generation. Input data are split into labeled and unlabeled datasets. In the E-step, pseudo-labels (  ) are generated for unlabeled data using the model's predictions ( *θ* ) with a fixed threshold ( *T* = 0.5). In the M-step, the model is refined by optimizing a combined loss function ( *L _total_* ) comprising supervised Dice Loss ( *L*_*L*_ ) for labeled data and unsupervised Dice Loss ( *L _U_* ) for pseudo-labeled data. The process iterates until convergence, yielding the final segmentation output.

Bayesian Pseudo Labels (SegPL) 33 is an SSL method particularly effective for small or noisy datasets. The method frames pseudo-labeling as an Expectation-Maximization (EM) algorithm, iteratively refining pseudo-labels and model parameters to improve segmentation accuracy ( Fig. 3 ).

*E-step*

In the E-step, pseudo-labels (  ) for unlabeled data ( *x _U_* ) are generated by estimating their posterior probabilities using the model's predictions ( *θ* ):



Here, *T* represents the threshold for determining pseudo-labels. In standard BPL, *T* is fixed (commonly 0.5). However, BPL can also employ variational inference to dynamically learn *T* , allowing for more adaptive thresholding in noisy datasets.

*M-step*

In the M-step, the pseudo-labels (  ) generated in the E-step are used to update the model parameters ( *θ* ) by optimizing a combined loss function over both labeled ( *X _L_* ) and unlabeled ( *X _U_* ) data:



where

is the unsupervised loss on pseudo-labeled data.
is the supervised loss on labeled data.
- *α* is a hyperparameter controlling the weight of the unsupervised loss.

#### nnU-Net

nnU-Net (no new U-Net) builds on the original U-Net architecture by automating the configuration process, including preprocessing, network architecture, training, and postprocessing. 34 35 This automation simplifies the setup, making it easier to train and deploy U-Net in new environments.

For this study, nnU-Net version 2 was used, which requires minimal metadata for training, thereby streamlining the setup process. Key metadata fields include the type of imaging modality, labels, and the number of training samples. Virtual environments were set up according to the model requirements before using the nnU-Net models.

### Study Design

Several factors were considered when designing this study for the two semisupervised methods: the small size and uniqueness of the datasets (two initial datasets), the large size of individual datasets (several GBs when zipped), very sparse labels, lack of a well-labeled region for validation/testing, and limited computing resources. Initially, only the two larger datasets (A and C) and an unlabeled dataset (Dataset D) were available; the smaller dataset (B) became available later.

The study design was informed by lessons from Oliver et al on evaluating SSL algorithms. 36 It involved comparing the semisupervised model architecture with a fully trained supervised version, as well as a transfer learning model, though this was not possible. Specifically, MisMatch, based on a U-Net architecture, was compared with nnU-Net, which achieves state-of-the-art performance across different segmentation tasks. 35 Additionally, the study varied the ratio of labeled to unlabeled data, as algorithms can be sensitive to this ratio. It is also important to report if the unlabeled data for training comes from outside the distribution used for training, as this can worsen performance. In this study, the unlabeled dataset came from the same distribution as one of the two training samples (IPF and PPFE).

Two study designs were devised to achieve the clinical utility of propagating labels for downstream analysis while working within these constraints:

Design 1: Use all initial available data for training (Dataset A + C) while performing a qualitative assessment on the unlabeled dataset. This approach aimed to provide sufficient data for meaningful insights and assess the generalizability of the model when trained on sparse labels, particularly in the PPFE/IPF labeled samples. Once dataset B became available, it was used to test and compare the performance of an optimized supervised method. The downside was the lack of a validation set for testing overfitting or hyperparameter tuning, which was considered less critical given the sparse labeling across all datasets.

Design 2: This approach aimed to optimize performance on a single distribution (PPFE) by focusing on the dataset with the highest label density. Dataset B was used for both validation and testing due to its similar distribution to Dataset A and the limited availability of labeled data. While this dual use of Dataset B introduces potential bias in reported performance metrics, it aligns with the study's objective to develop a model that performs well on a consistent data distribution. This focus on overfitting to a single distribution was deliberate to facilitate a **human-in-the-loop framework** , where human reviewers could iteratively correct predictions and retrain the model efficiently. Dataset C, containing both PPFE and IPF, was excluded from this design to avoid confounding effects from a mixed distribution and sparse annotations, which could reduce the model's ability to learn effectively. The limitations of this design, including potential overestimation of performance on Dataset B, are acknowledged, and qualitative assessments were prioritized over quantitative metrics.

These designs aimed to balance the need for data sufficiency, model generalizability, and the constraints of label sparsity and computational resources. Of note for the supervised method for comparison, as nnU-Net does its own preprocessing, Datasets A and C were given to train and Dataset B was used for test. All three study designs are summarized in Table 1 .

**Table 1:** Dataset usage in different training designs

Dataset	SSL Study Design 1	SSL Study Design 2	Supervised learning
A	Training	Training	Training
B	Test	Validation/Test	Test
C	Training	Not used	Training
D	SSL training	SSL training	N/A

Abbreviation: SSL, semisupervised learning.

### Data Preprocessing

#### Semisupervised Models

The two larger datasets initially available consisted of 2D images, while the semisupervised models required 3D volumes. To convert the .tiff files into 3D volumes, images and labels were loaded into lists and then converted into NumPy arrays, significantly reducing processing time. Images were cropped to remove non-tissue areas, and 3D patch sizes of 128, 256, and 512 pixels were used to manage memory constraints. Overlapping patches were employed to maintain spatial relationships and ensure all data was utilized.

Unlabeled data underwent equivalent preprocessing, including normalization, standardization, and conversion to NIfTI files. Data augmentation was performed on the fly, with MisMatch using random cropping and SegPL using random contrast, zoom, orthogonal slicing, and cropping.

#### nnU-Net

For nnU-Net, .tiff files were collated into 3D volumes in the NIfTI format, named, and filed according to nnU-Net requirements. The data was processed using the plan and preprocess function for preprocessing, fingerprint extraction, and experiment planning.

Models are summarized in Table 2 .

**Table 2:** Summary of semisupervised and supervised model details

Model	Architecture	Key technique	Loss function	Training features
MisMatch	U-Net with dual decoders	Consistency regularization via feature perturbation	Training Dice Loss + MSE	Positive/Negative feature attention
SegPL	EM-based pseudo-labeling	Dynamic thresholding for pseudo-labels	Dice Loss	Confidence-based label selection
nnU-Net	Self-configuring U-Net	Automated pipeline optimization	Dice Loss	Autogenerated preprocessing and postprocessing

### Hyperparameter Optimization

Hyperparameter tuning was limited due to scarce validation data. For MisMatch ( Supplementary Table S1 [available in the online version]), α (the weight for the unsupervised loss function) and batch size were optimized for regularization and training stability. For SegPL ( Supplementary Table S2 [available in the online version]), the ratio of unlabeled to labeled data was optimized, as it is a critical parameter. 36

### Data Analysis

Analyzing the results presents several challenges. First, without fully annotated samples, a reliable test set for quantitative analysis is unavailable. Predictions might be incorrectly classified as false positives due to the absence of ground truth labels for certain vessels. Second, the scarcity of labeled data means that using any for testing would further reduce the training set, potentially degrading model performance.

The ideal testing method would involve reconstructing the entire volume to assess structural connectivity. However, given the limited data, only one volume at most could be used for testing, making statistical comparisons impractical due to insufficient observations. Comparing individual chunks before reconstruction might not be meaningful, as sparse labeling in some chunks would distort the metrics.

Filtered test set images ensuring a minimum percentage of labeled data per chunk could facilitate quantitative comparisons. However, this would alter the original image and may not accurately reflect model performance. Such filtering could lead to overestimation of performance, particularly in terms of positive predictive value, by excluding complex structures that mimic vessels. Consequently, while some basic metrics were observed, statistical tests were deemed inappropriate, and a qualitative review was preferred in these initial stages.

Finally, as outlined in Study Design 2, Dataset B served as both validation and test data. This choice was driven by the limited availability of labeled data and the need to focus on a single distribution (PPFE). While this approach emphasizes model performance on a consistent dataset, it also highlights the limitations of quantitative metrics due to sparse annotations and potential overlap in validation and test data usage.

## Results

### Dataset Evaluation

Three datasets and their corresponding label maps were evaluated, consisting of two classes: vessels (label) and non-label (everything else, including biopsy tissue contents and background). The exact number of vessels labeled in each sample is unknown due to variations in vessel quantity and labeling levels. The total volume of labeled data and visual inspection served as surrogate markers, as shown in Table 3 . Dataset C was sparsely labeled with only 0.1% of all available voxels being labeled (compared with 0.9% for Dataset A and 1.7% for Dataset B) and on visual inspection these were all small vessels. Dataset B, had the greatest percentage of available voxels labeled, though it was the smallest dataset with only 220 slices and therefore had almost five times fewer labeled voxels than Dataset A (12.5 million vs. 56.6 million labeled voxels), though these were mostly of larger vessels. Dataset A had the most labeled vessels due to its larger volume, with just under 1% of the volume labeled.

**Table 3:** Percentage of labeled volume within the total imaged volume for each of the three labeled datasets

Dataset	Pathology	Slices	Total number of labeled pixels within volume	Total number of pixels (labeled and unlabeled) within volume	Percentage of total volume with labeled pixels
A	PPFE	1,863	56,665,263	6,188,038,598	0.9157%
B	PPFE	220	12,491,944	731,132,380	1.7086%
C	PPFE + IPF	960	3,204,402	3,146,478,048	0.1018%
D	PPFE	1,300	N/A	N/A	N/A

Abbreviations: IPF, idiopathic pulmonary fibrosis; PPFE, pleuroparenchymal fibroelastosis.

### Label Quality and Image Review

Labels were inspected for quality, with images labeled approximately every five slices and interpolated in between. Vessels were defined as everything inside the outer walls, including the lumen. Visual inspection revealed significant variation in label quality, from partial to full inclusion of vessels, complicating the network's task of identifying label class features. The images themselves differed significantly. Normally, a lung image shows a thin pleural layer at the edge, with the lung appearing as a black background with a web-like overlay of terminal acini and airways interspersed with vessels. However, the PPFE sample showed collapsed and fibrosed tissue with little “black” lung tissue, while the PPFE and IPF samples showed some airways. Pixel intensities of vessel walls were similar to the background, though often with a visual boundary. Histograms of image intensities for labeled and non-labeled areas showed significant overlap ( Supplementary Figs. S1 and S2 [available in the online version only]). In the PPFE datasets, two overlapping histograms were observed, with peaks representing vessel walls and lumens. The PPFE and IPF datasets showed only one clear peak, likely due to obscured secondary peaks from the background pixel distribution. Dividing main volumes into smaller volumes showed varying degrees of overlap, reflecting differences in tissue, vessel lumen, vessel wall, and background.

### Training Challenges and Improvements

Initially, the MisMatch algorithm was trained on the 3D 512-pixel patches. Larger patches typically yield better performance by reducing the loss of contextual information. The initial experiment, using baseline hyperparameters and a batch size of 2, produced noisy, non-converging training due to the random 3D 96-pixel crops from the data generator often lacking labels. To address this, images were filtered for labels at different levels (>0, 1, 2.5, and 5% of the image volume) to ensure consistent labeled data. Experiments focused on smaller patch sizes (128 and 256 pixels), closer to the cropped patch size, improving training speed and model performance, reducing training time to days. Validation sets were used to monitor overfitting, which was not a significant issue given the sparse labeling. The same strategy was applied to the SegPL method. Training time per model was substantial, with GPU and memory requirements as limiting factors. Training curves were monitored, and models were cut when the curve began to flatten to balance performance and time efficiency. Supplementary Tables S3 S4 S5 S6 (available in the online version) detail the training results. SegPL generally achieved more stable and better metrics compared with MisMatch. Smaller patches trained faster, likely because there was less chance of empty or near-empty labels. Increasing labeled samples and batch size, and decreasing α (reducing regularization) improved SegPL's performance. Validation curves showed models often began to “overfit” within the first 10 to 20,000 iterations, though this was not necessarily when the best segmentation maps were produced visually due to incomplete labeling. Larger patch sizes during validation were not possible due to memory constraints. Supplementary Table S7 (available in the online version) shows nnU-Net results, which performed well on unpatched data with a DICE score of 0.75 on the full volume. However, performance declined once the data were patched and normalized. Postprocessing for nnU-Net, still under development, showed that simple thresholding was ineffective as it removed tiny vessels.

### Segmentation Comparison

**Fig. 4:**
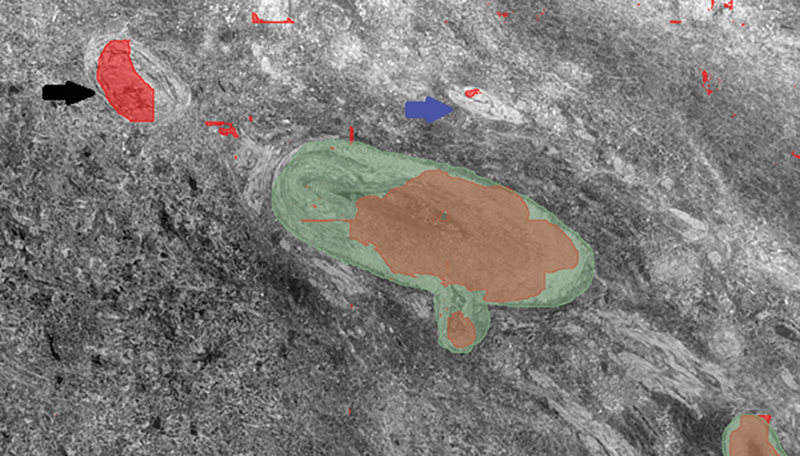
MisMatch segmentation overlay (red) on incomplete ground truth labels (green) from dataset B using Design method 1. Of note, a vessel in the top left-hand corner (black arrow) was not labeled in the ground truth, as well as a vessel in the middle (blue arrow). There is incomplete labeling of the ground truth. There is partial labeling of the two noted vessels not in the ground truth, with minimal additional false positive labels.

Example segmentations from the five different strategies ( Figs. 4 5 6 7 8 ) showed similar results, with false positives needing removal. nnU-Net segmented larger and medium-sized vessels more effectively, while MisMatch and SegPL also segmented smaller vessels. MisMatch had slightly fewer false positives. Single distribution training with a validation set produced slightly better segmentations for MisMatch (which may be expected as Dataset B was used for validation), though there was no significant difference between the two design methods when tested with SegPL. This also highlights the limitation of relying solely on metrics in the setting of incompletely labeled data, as they may not accurately reflect segmentation quality.

**Fig. 5:**
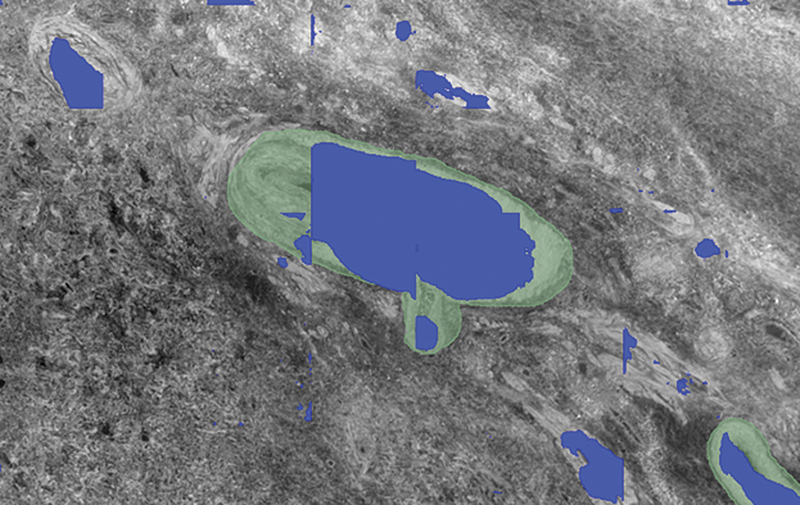
MisMatch segmentation overlay (blue) on incomplete ground truth labels (green) from dataset B using Design Method 2. Incomplete labeling of larger ground truth vessel. More complete labeling of the two noted vessels not in the ground truth compared with the other training strategy, as well as other small vessels, however further additional false positive labels.

**Fig. 6:**
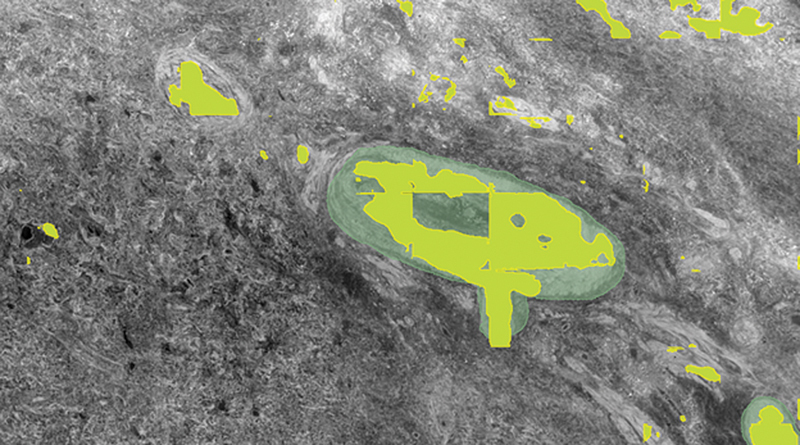
SegPL segmentation overlay (yellow) on incomplete ground truth labels (green) from dataset B using Design Method 1. There is a labeling of the two noted vessels not in the ground truth, as well as other small vessels, but with some additional false positive labels, more than seen in MisMatch.

**Fig. 7:**
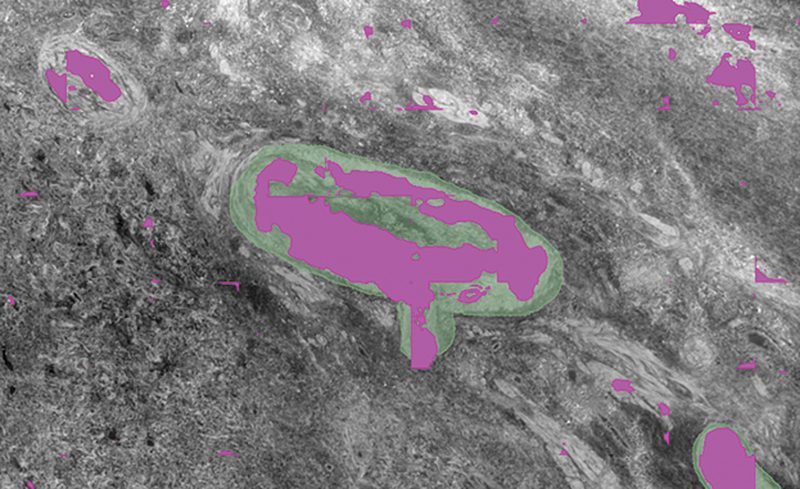
SegPL segmentation overlay (pink) on incomplete ground truth labels (green) from dataset B using Design Method 2. Prediction from SegPL with validation (pink). There is labeling of the 2 vessels not in the ground truth, as well as other small vessels, but with some additional false positive labels.

**Fig. 8:**
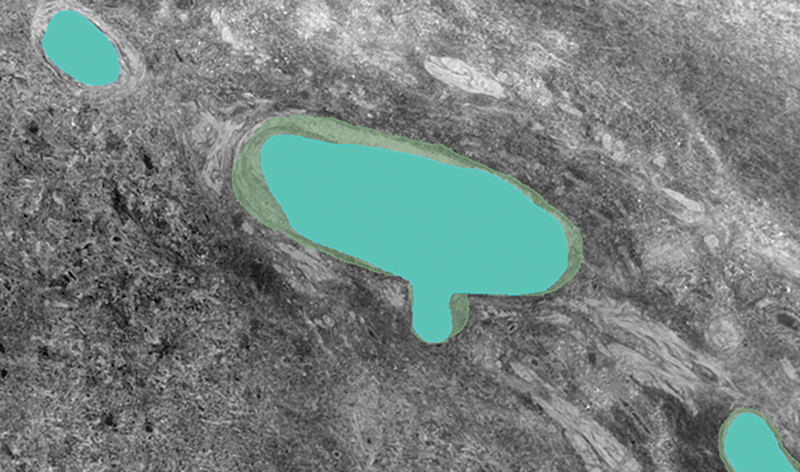
nnU-Net segmentation overlay (blue) on ground truth labels (green) from dataset B. Good segmentation of the ground truth label and of the small vessel in the top left-hand corner which was not originally labeled. However, smaller vessels are not labeled.

## Discussion

This study has shown promise for SSL models for vascular segmentation in HiP-CT datasets, particularly when compared with the state-of-the-art supervised method for smaller out-of-distribution vessels. However, supervised learning provided more consistent segmentations of the majority label phenotype of vessels. Several limitations were noted at the onset, not least the lack of labeled data which was discussed as a motivation for this piece of work in the introduction section. However, unlike traditional imaging techniques such as CT where vessels typically appear as tubular structures, HiP-CT at the microscopic level exhibits a wider variation in structural appearances. This, combined with the large volume of data in each stack, made potential noise in the annotated data a significant limitation. Limited labels also risked selection bias, in which the predominantly labeled vessels are preferentially segmented, as seen with nnU-Net, and hence for a supervised approach, a more representative labeled sample may help as well as highlighting the need for “human-in-the-loop” validation. Model bias must also be considered, as pseudo-labeling methods often assume balanced class distributions, which was not the case in this sparsely labeled data. Proper model validation and implementation strategies in addressing class imbalance in pseudo-labeling are essential to mitigate these biases and prevent downstream inequalities. 37

To further improve the study, comparisons with transfer learning methods, as suggested by Oliver et al could be undertaken. 36 However, finding a model trained on a similar 3D task is challenging. Improvements to preprocessing pipeline could include focusing on training with a single distribution of data to explore the potential for an active learning approach, though no significant advantage was found here. Also, favoring a model with a tendency for false positives could be preferable, as it is quicker to remove false positives than to add new labels. Other improvements noted from the results would be to completely remove background areas especially as small patch sizes produced significant artifacts and even increase the number of non-expert labeled samples, as those have been shown to lead to accurate segmentations. 38 To address overlabeling (false positives) in the pseudo-labeling samples, a histogram-based attention mechanism, could be beneficial, especially since histogram overlap is less pronounced in patches. 39 Finally, in postprocessing, dealing with connectivity remains a challenge. A single threshold is ineffective as some areas are larger than vessels.

Vessel segmentation on sparsely labeled data presents unique challenges, particularly when comparing various SSL methods across different imaging modalities. Most literature focuses on 2D retinal imaging, which complicates direct comparisons with HiP-CT data, not least because the 3D data create computational and algorithmic challenges. Additionally, retinal imaging benefits from abundant data, even if unlabeled, whereas HiP-CT is an emerging technology with fewer samples from diverse sources. The morphological and structural differences between vascular structures in conventional retinal, cerebral, etc., imaging that generally resemble tubular structures are very different from those observed in HiP-CT images, which more closely resemble histopathological images but in 3D volumes.

Reviewing literature on SSL vessel segmentation, two notable studies, Hou et al (2021) and Lin et al (2023) focus on the Mean Teacher Method, which employs a student-and-teacher network paradigm. 39 40 Both networks start with the same random or pretrained weights. The student network is trained on labeled data, calculating a supervised loss, while unlabeled data are passed through both networks, and a consistency loss is calculated between them. The student's weights are updated based on these losses, while the teacher's weights are updated through an exponential moving average of the student's weights, providing regularization, and reducing the likelihood of overfitting. Hou et al integrate adversarial and consistency regularization within a GAN-based framework, improving generalization by making the discriminator's task more challenging and forcing the network to improve. Results on retinal datasets show slightly better sensitivity, but slightly reduced specificity compared with other state-of-the-art models. The clinical utility of this increased performance, the computational cost, and generalizability to other tasks remain unexplored.

Lin et al applied SSL via a teacher–student network using Swim-U-Net as the backbone. The teacher network is trained on labeled data to minimize cross-entropy, dice similarity, and boundary loss, producing predictions that serve as pseudo-labels for the student network. Pseudo-labeling often leads to oversegmentation, so they employ “adaptive histogram attention” to minimize this, focusing the model on vessel areas. Tested in brain vessel images, the network demonstrates lower surface error compared with nnU-Net and Cross Pseudo Supervision, indicating better performance in labeling unlabeled data. However, high memory usage suggests significant computational costs, which may not be feasible for large HiP-CT datasets.

Another study proposed a “hierarchical segmentation network,” using a pseudo-label approach to leverage unlabeled data. 41 It initially uses labeled data to train a posterior network, where the posterior distribution of the image is learned from its label. This, in turn, is used to train a prior network, with K–L divergence loss ensuring minimal difference between the prior and posterior networks. This prior network then obtains pseudo-labels on unlabeled data, with a confidence threshold above which a pseudo-label is retained. The new pseudo-labeled data are then used iteratively to train the segmentation network. The performance of the model is evaluated on both 2D retinal images and 3D liver CT images, showing improved accuracy on 3D images compared with other methods, though with reduced sensitivity (61.5% vs. 70.0%) indicating lower effectiveness in detecting vessels, which are the minority class. 42 43 Notably, the sensitivity for 3D vessel segmentation (61.5%) is lower than that for 2D retinal imaging (79%), highlighting the challenges posed by 3D datasets.

These studies indicate various techniques applied to relatively limited data types, highlighting that vessel segmentation in sparsely labeled datasets is still a valid area of research.

## Clinical and Public Health Implications

The clinical implications of this work are significant, demonstrating the potential to achieve vessel segmentation in HiP-CT data. This advancement potentially paves the way for the study of disease processes that are limited by existing imaging techniques. Specifically, if vascular imaging biomarkers could be identified using HiP-CT before the onset of fibrosis, it could potentially enable the mapping of these biomarkers onto current clinical CT scans. This would provide surrogate biomarkers for pharmaceutical interventions, targeting disease processes before irreversible damage occurs.

This study offers initial insights into the challenges of vessel segmentation in HiP-CT and presents methodologies to address them. By addressing the current limitations and leveraging advanced SSL models, this work paves the way for improved diagnostic tools and early detection methods. These advancements can significantly impact patient outcomes by facilitating early treatment and potentially slowing the progression of chronic lung diseases like IPF and PPFE. This research also contributes to a broader understanding and application of vessel segmentation in HiP-CT, which can extend to other diseases where microvascular changes are critical.

## Conclusion

Overall, this study has shown promise in using SSL models for vascular segmentation in HiP-CT datasets, particularly when compared with the state of the art supervised method for smaller out-of-distribution vessels, though supervised learning provided more consistent segmentations of the majority label phenotype of vessels (large). However, several major obstacles remain, including the need for improved annotation processes, better model optimization, and effective postprocessing strategies, not forgetting the challenges of dealing with vast quantities of data. Addressing these challenges will be crucial for advancing the application of SSL in medical imaging.
