# Supplementary material for: Harnessing Advanced Machine Learning Techniques for Microscopic Vessel Segmentation in Pulmonary Fibrosis Using Novel Hierarchical Phase-Contrast Tomography Images
Source: Methods Inf Med. 2025 May 9;63(03-04):97–108. doi: 10.1055/a-2540-8166 (PMC12133326; doi:10.1055/a-2540-8166)
Supplement: Supplementary file 1 — Supplementary Material [file 10-1055-a-2540-8166-s24020005.pdf]

Supplementary Appendix

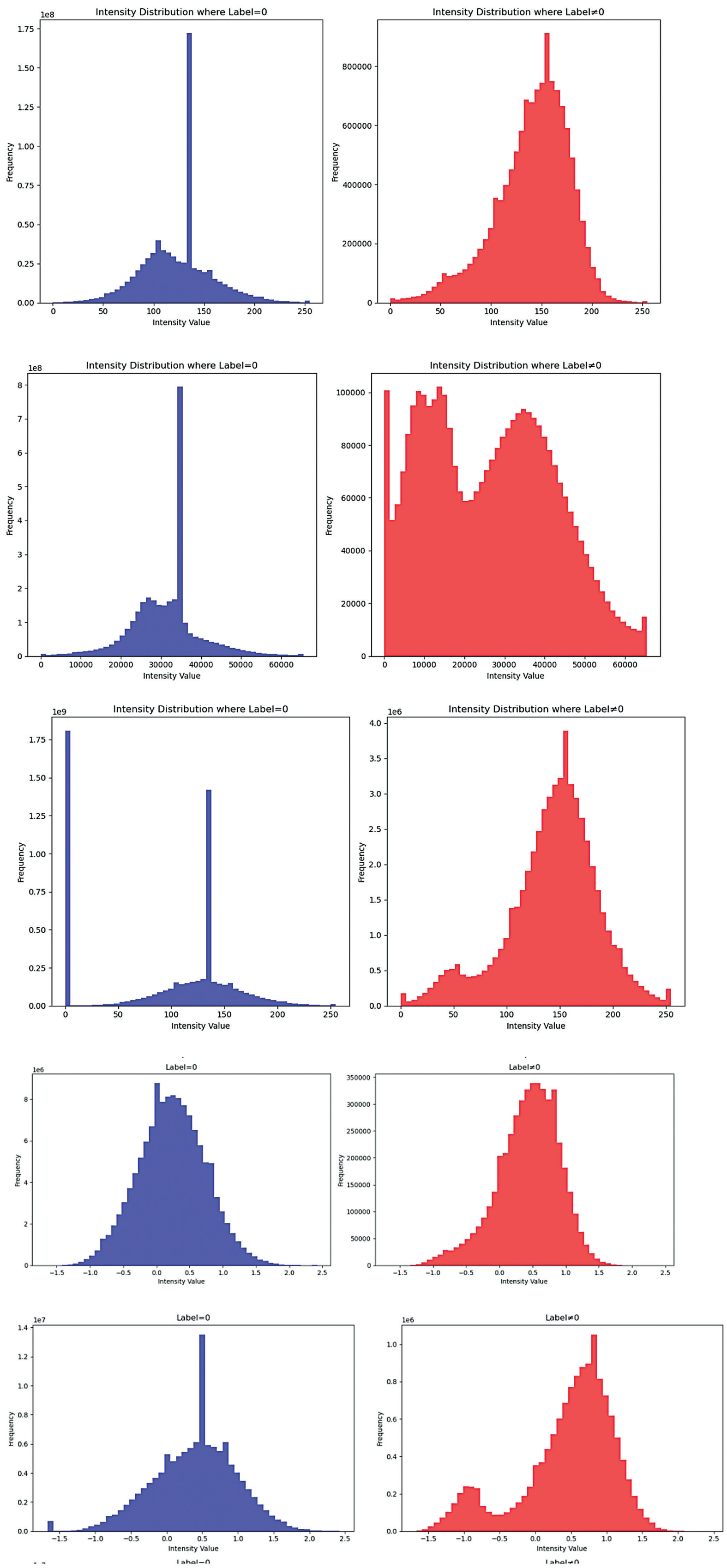

Supplementary Fig. S1 Separate patches where background intensities and vessel intensities defined by labeled areas in the patch have been compared. Label $\neq 0$ is vessel and Label $= 0$ is everything else including background. There is a subtle difference in peaks though significant overlap.

 © 2025. The Author(s).

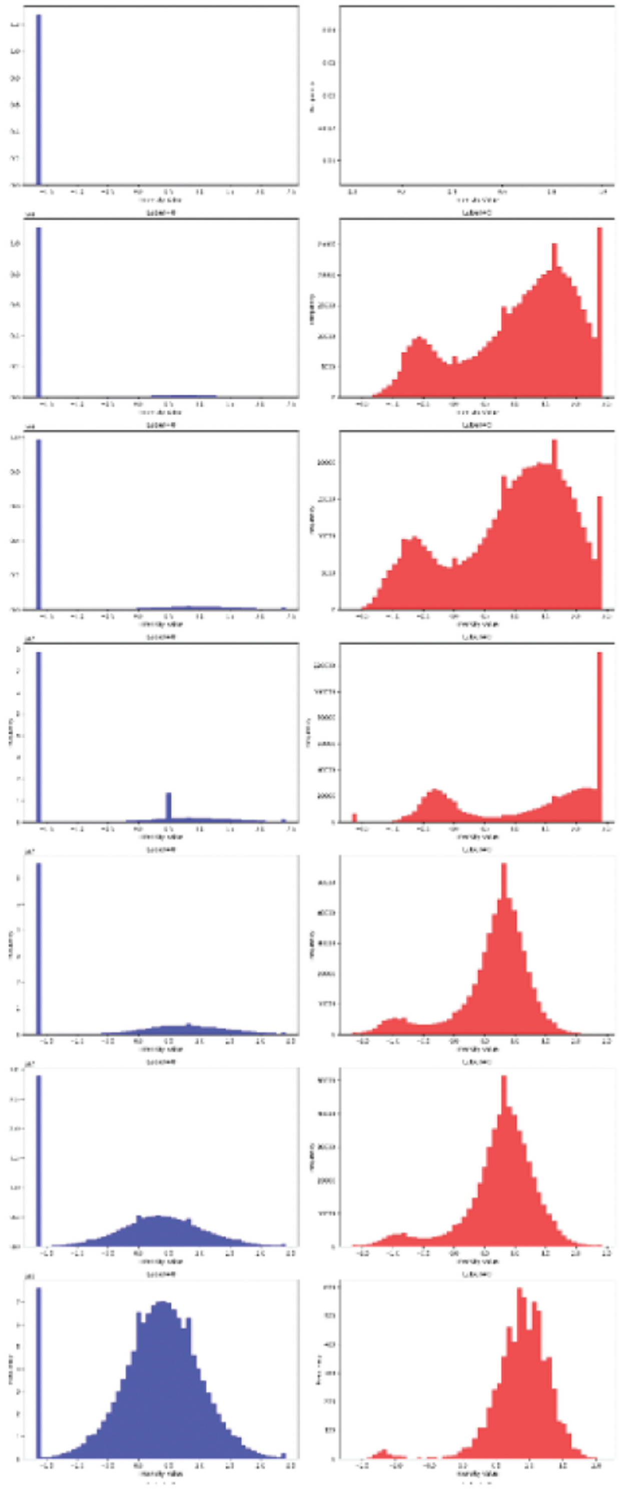

Supplementary Fig. S2 Separate patches where background intensities and vessel intensities defined by labeled areas in smaller patches (size 128×128) have been compared. Label $\neq 0$ is vessel and Label $= 0$ is everything else including background. In the 10 smaller patches, the differences in the distribution of the vessels become more apparent.

 © 2025. The Author(s).

Supplementary Table S1 Example hyperparameters to tune for MisMatch model

Hyperparameter	Explanation	Value in paper
Batch size	Batch size of labeled volumes.	1
Optimizer	This specifies the algorithm used for weight optimization across nodes	Adam
Learning rate		2e-5
α	Consistency regularization weight	0.002
epochs	Number of epochs	50

Supplementary Table S2 Example hyperparameters to tune for SegPL (Bayesian Pseudo Label)

Hyperparameter	Explanation	Value in paper
Batch size	Batch size of labeled volumes	2
Optimizer	This specifies the algorithm used for weight optimization across nodes	Adam
Learning rate		0.001
Temp	Temperature scaling on output	1
Batch_u	Set to 0 for supervised setting	2
Pri_mu	Mean of prior	0.7
Pri_std	Standard deviation of Prior	0.15
α	Weight on the unsupervised part if semisupervised learning is used	1
β	Weight for pseudo-supervision loss	1
warmup	Ratio between warmup iterations and total iterations	0.1
Warmup_start	Ratio between warmup starting iteration and total iterations	0.4

 © 2025. The Author(s).

Supplementary Table S3 Table showing experiments performed on SegPL, without validation sets on Datasets A + C

	train iou ema (smoothing 0.60)	train seg loss (smoothing 0.60)	epoch	train time (days)
Patch size 128				
t0	0.8612	0.2032	37036	2.309
t1	0.939	0.0824	31409	2.311
t2.5	0.936	0.06175	35095	2.27
t5	0.9637	0.04149	27016	2.305
α 2.0 batch 8	0.9742	0.03204	31806	5.625
α 0.9 batch 8	0.9698	0.03368	29376	5
t2.5				
α 0.1	0.954	0.05073	49380	2.315
α 0.5	0.9554	0.0686	33150	2.312
α 2.0	0.9499	0.0683	33483	2.312
Batch 4	0.9641	0.0694	22891	2.778
Batch 8	0.9666	0.0633	13409	2.775
Primu 0.5	0.9656	0.0871	31174	2.312
Primu 0.7	0.943	0.0598	32104	2.312
Patch size 256				
t0	0.6801	0.4355	10079	2.298
t1	0.8305	0.2578	26820	4.617
t2.5	0.8714	0.2187	22100	4.617
t5	0.8798	0.2295	14882	4.617
Patch size 512				
t0	0.5251	0.6273	1297	2.29
t1	0.6266	0.5432	1539	2.304
t2.5	0.6658	0.4908	2819	4.617
t5	0.8049	0.397	4364	4.617

The left-hand column represents the datasets by patch size (128, 256, 512) and is further divided by the percentage of thresholding (t). Alpha, batch, and primu refer to different hyperparameters.

 © 2025. The Author(s).

Supplementary Table S4 Table showing experiments performed on SegPL, with validation sets on Dataset A

	best train iou	best validation iou	train seg loss (smoothing 0.60)	epoch	train time (days)
128					
t0	0.8755	0.6099	0.2032	26596	2.315
t1	0.9394	0.709	0.061	50000	4.2
t2.5	0.9751	0.7167	0.0488	50000	4.6
t5	0.9608	0.702	0.0569	18514	1.197
t2.5					
α 0.5	0.9599	0.7045	0.0463	50000	2.312
α 2.0	0.9499	0.7061	0.0571	33750	2.312
Batch 4	0.9601	0.7115	0.0618	23744	2.775
Batch 8	0.9636	0.7117	0.0556	25195	4.6
Primu 0.5	0.9656	0.7095	0.0592	33000	2.312
Primu 0.7	0.9457	0.7003	0.0598	27230	2.312
256					
t0	0.6688	0.4362	0.4842	6742	2.3
t1	0.8919	0.5788	0.301	10827	5.2
t2.5	0.8215	0.6411	0.275	10500	5.2
t5	0.8773	0.634	0.2208	11085	5.2

The left-hand column represents the datasets by patch size (128, 256) and is further divided by the percentage of thresholding (t). Alpha, batch, and primu refer to different hyperparameters.

Supplementary Table S5 Table showing experiments conducted on MisMatch, without validation sets on Datasets A + C

	loss	loss_seg	loss_seg_dice	epoch	train time (hours)
Patch size 128					
t0	0.4705	0.04732	0.6182	51720	2.302
t1	0.3805	0.05934	0.5355	9863	10.12
t2.5	0.3176	0.06803	0.4779	80272	6.5
t5	0.3039	0.08395	0.4032	29614	22.13
t2.5					
con 0.5	0.4121	0.0907	0.4358	20668	12.65
con 5.0	–	–	–	–	–
Batch 8	0.2617	0.04731	0.398	48197	2.308
Batch 16	0.3368	0.07866	0.5027	28268	2.308
lbl 4	0.2683	0.02773	0.5051	56139	2.312
lbl 8	0.2923	0.0378	0.4966	33539	2.687
Patch size 256					
t0	0.4812	0.01531	0.7267	77640	4.6
t1	0.4049	0.02524	0.4709	117356	5.8
t2.5	0.3166	0.03799	0.5539	80192	7
t5	0.3605	0.03999	0.5457	45747	2.3

The left-hand column represents the datasets by patch size (128, 256) and is further divided by the percentage of thresholding (t). Con (consistency), batch, and lbl (the number of labeled) data in the dataset, refer to different hyperparameters.

 © 2025. The Author(s).

Supplementary Table S6 Table showing experiments conducted on MisMatch, with validation sets on Dataset A

	train loss	validation loss	train loss_seg	validation loss_seg	train loss_seg_dice	epoch	train time (hours)
Patch 128							
t5	0.217	0.2904	0.2655	0.4443	0.3139	120000	3.44
t2.5							
con 0.5	0.3094	0.2787	0.2148	0.4504	0.296	na	na
con 5.0	0.26	0.285	0.2496	0.4574	0.02297	80000	na
lbl 4	0.2903	0.2753	0.2489	0.5417	0.3016	70000	na
lbl 8	0.2922	0.3001	0.2762	0.5325	0.3132	22500	2.164

The left-hand column represents the datasets by patch size (128) and is further divided by the percentage of thresholding (t). Con (consistency) and lbl (the number of labeled) data in the dataset, refer to different hyperparameters. Further experiments were not possible with larger patch sizes due to the lack of memory.

Supplementary Table S7 nn-UNet performance on Dataset B for differently prepared datasets

Training datasets (A + C)	IoU	Dice	FN pixels	FP pixels	TN pixels	TP pixels	Predicted label pixels	Total label pixels
Full volumes	0.6057	0.7544	2846910	3431312	715209124	9645034	13076346	12491944
128 patches	6.40E-07	1.28E-06	12491936	35	718640401	8	43	12491944
256 patches	0	0	12491944	0	718640436	0	0	12491944
512 patches	0	0	12491944	0	718640436	0	0	12491944
128 patches with 1% threshold	0.0020	0.0041	12466076	162162	718478274	25868	188030	12491944
256 patches with 1% threshold	0.0147	0.0290	6821466	372406820	346233616	5670478	378077298	12491944
512 patched with 1% threshold	0.0048	0.0097	12428839	434954	718205482	63105	498059	12491944

Abbreviations: IoU, Intersection over Union; FN, False negative; FP, False positive; TN, True negative; TP, True Positive.

 © 2025. The Author(s).
